# Comment on “Short- and long-term risk stratification in acutely ill medical patients by implementing D-dimer in the emergency setting – A prospective cohort study”

**DOI:** 10.1016/j.ahjo.2026.100962

**Published:** 2026-10-14

**Authors:** R. Jayanthi, Gadi Madhavi, A. Damodhara Velayudhan, N. Srimathi

**Affiliations:** a Obstetrics and Gynaecology, Meenakshi Medical College Hospital & Research Institute, Meenakshi Academy of Higher Education and Research, Enathur, Kanchipuram, Tamil Nadu, 631552, India; b Arulmigu Meenakshi College of Nursing, Meenakshi Academy of Higher Education and Research, Chennai, Tamil Nadu, India

**Keywords:** D-dimer, Emergency medicine, Risk stratification, Acutely ill patients, Short-term outcomes, Long-term outcomes

Dear Editor,

Kornfehl et al. [1], Measured D-dimer levels in 1035 emergency patients who were not selected by any criteria. Study findings show that a high D-dimer level can predict death at both 30 days and at 360 days. Study design was very clean because researchers tested every patient and then kept the results that were not requested by the treating team. Subgroup counts match perfectly. Mortality numbers do not.

In the baseline summary the column for D-dimer shows 13 deaths at 30 days 4 deaths at 180 days and 1 death at 360 days. Cumulative mortality cannot decrease [2]. The column for D-dimer lists 23 deaths at 30 days 37 deaths at 180 days and 65 deaths at 360 days which appears correct. Thus one column contains errors. The numbers 13 and 1 have been swapped. The rows confirm this: when the numbers are restored to 23 and 1 the total deaths at 30 days become 24 and when the numbers are restored to 65 and 13 the total deaths at 360 days become 78. The main text includes the corrected values. Only the summary contains mistakes. The summary is the first thing a reader looks at.

The one-year count does not settle. Study results report 80 deaths, which's 7.7% of the cohort and the abstract repeats 7.7%. The summary however reports 78 deaths, which's 7.5%. Adding the discharged and admitted strata yields 22 deaths, 3 deaths, 43 deaths and 10 deaths, which together equal 78 deaths. Two deaths appear in the narrative. Are not mentioned elsewhere in the paper.

The subset that matters most also has percentages. Among the 284 patients whose physicians actually ordered the dimer test 165 patients had elevated D-dimer and mortality among these patients is reported as 1.8% (five patients) and 8.1% (23 patients) [3]. Five out of 165 equals 3.0% and 23 out of 165 equals 13.9%. Both printed percentages are calculated using 284 as the denominator the total subset. This analysis is closest to clinical practice and the 30-day difference in this subset fails to reach significance (*p* = 0.067).

That *p*-value deserves attention than it receives. In the cohort the 30-day comparison shows 23 deaths versus one death. The area under the curve of 0.82 and a sensitivity of 91.7% both depend on the placement of that death.

Two outcomes are missing. The abstract states that survival was analysed at 30 days, 90 days and 360 days. The 90-day outcome is not mentioned else [4]. Instead the paper mentions 180 days an outcome that was never pre-specified in the methods.

Elsewhere the paper gives a survival time of 764 days for the cohort and 892 days for the routine subset. Because 7.5% of patients are dead median survival cannot be estimated. Both numbers represent follow-up durations.

Smaller issues also exist. The paragraph in the plausibility section that explains coagulation and fibrinolysis appears twice verbatim, a few sentences apart. Age-adjusted mortality among admitted patients with D-dimer is 15%, based on 15 deaths, which would require a denominator of 100 in a stratum of about 190 patients. The age-adjusted cut-off reference is listed under different numbers. Additionally the registry study cited for admission mortality is cited again for a finding about imaging in suspected SARS-CoV-2 infection, which's not what that study reports.

The prognostic argument is strong. The discharged patients, with elevated D-dimer are the part I would build upon. The arithmetic must first agree with itself.

## CRediT authorship contribution statement

**R. Jayanthi:** Writing – review & editing, Writing – original draft, Visualization. **Gadi Madhavi:** Conceptualization, Data curation, Formal analysis. **A. Damodhara Velayudhan:** Writing – review & editing, Writing – original draft, Visualization. **N. Srimathi:** Writing – review & editing, Writing – original draft, Visualization.

## Ethical approval

Not applicable (commentary on previously published research).

## Declaration of Generative AI and AI-assisted technologies in the writing process

Generative AI tools were used solely for language refinement and formatting assistance. All scientific interpretation, critique, and conceptual analysis were independently developed by the authors.

## Funding

None.

## Declaration of competing interest

The authors declare no conflicts of interest.

## Data Availability

No new data were generated or analysed in this work.
